# Susceptibility to metazoan parasite infection in amphimictic diploid and induced triploid tench (*Tinca tinca* L., 1758): the role of parasites in fish aquaculture

**DOI:** 10.3389/fvets.2025.1686708

**Published:** 2025-11-13

**Authors:** Andrea Šimková, Martina Dávidová, Pavel Hyršl, Michal Janáč, Martin Flajšhans, Markéta Ondračková

**Affiliations:** 1 Faculty of Science, Department of Botany and Zoology, Masaryk University, Brno, Czechia; 2 Faculty of Fisheries and Protection of Waters, South Bohemian Research Center of Aquaculture and Biodiversity of Hydrocenoses, University of South Bohemia in České Budějovice, Vodňany, Czechia; 3 Faculty of Science, Department of Experimental Biology, Masaryk University, Brno, Czechia; 4 Institute of Vertebrate Biology of the Czech Academy of Sciences, Brno, Czechia

**Keywords:** tench, ploidy, metazoan parasites, fish condition, physiology, immunity

## Abstract

**Introduction:**

Artificial induction of polyploidy in fishes is a widely used method in commercial aquaculture due to its economic potential and its association with changes in cell morphology and physiology that can significantly affect individual fitness. Using tench (*Tinca tinca*, Cyprinidae), a fish species extensively farmed in European aquaculture, we investigated differences in parasite susceptibility between triploid and diploid fish and analyzed the potential effect of metazoan parasite infection on fish condition, physiology, and health status.

**Methods:**

Amphimictic diploid and induced triploid specimens collected from a breeding pond were examined during four sampling events over the course of one year, focusing on the presence of metazoan parasites and selected fish condition, physiological, and immune parameters.

**Results:**

Diploids generally exhibited a higher overall parasite load than triploids throughout the year; however, this difference was statistically significant only in summer, coinciding with the extremely high abundance of the tench-specific *Asymphylodora tincae*. Host ploidy appeared to influence susceptibility or resistance to particular parasite species. While amphimictic diploid tench showed higher susceptibility to adult trematode *A. tincae*, triploid tench were more parasitized by the larval cestode *Valipora campylancristrota*. No difference in abundance of monogenean *Gyrodactylus tincae* was observed between amphimictic diploid and induced triploid specimens. Other parasites were relatively rare in both groups. Although no clear association between parasite infection and the measured physiological or immune parameters was found, significant negative correlations were more frequently observed in diploid fish than in triploids.

**Discussion:**

The differences in parasite infection between triploid and diploid tench and the associations between parasite load and condition- and health-related traits may be explained by (1) presumed higher heterozygosity in triploids, (2) physiological differences related to cell size and number of cells in key organs and tissues, (3) variation in feeding performance, and (4) host–parasite coevolutionary interactions.

## 1 Introduction

Polyploidization, the multiplication of the whole chromosome complement, is a widespread phenomenon in plants and occurs sporadically in animals. Polyploidy represents a special type of mutation and can arise through several mechanisms, such as genomic doubling, gametic nonreduction, or polyspermy (1). It is consistently associated with changes in cell morphology and physiology, which can significantly affect individual fitness (2, 3). In fish, spontaneous polyploidy is known to occur both within species (autopolyploidy) and across species (allopolyploidy) [e.g., Leggatt and Iwama (4), Cunha et al. (5), Havelka et al. (6), and Schreier et al. (7)]. Polyploidy may confer advantages in certain environments, such as increased stress tolerance or altered ecological interactions, resulting in novel traits and enhanced adaptation to new conditions.

In fish selective breeding biotechnological methods, including chromosomal manipulations such as induction of polyploidy, gynogenesis, androgenesis, or sex reversal, have been widely applied (8, 9). In particular, the artificial induction of polyploidy in fishes, most commonly aimed at producing triploid forms, has become an economically attractive and extensively used method in commercial aquaculture. The primary motivation for inducing triploidy in aquaculture is the expectation of beneficial traits, especially enhanced growth performance. This is largely attributed to the sterility of triploids, which prevents the growth depression typically associated with sexual maturation of diploids. Additional advantages include reduced sexual and territorial behavior, leading to lower stress levels and decreased energy expenditure, as well as increased survival in cases where reproduction is associated with elevated mortality of diploids (10, 11). Previous studies have confirmed that diploid and triploid fish differ in growth, survival, slaughter value, and flesh composition [e.g., Piferrer et al. (10), Flajšhans et al. (11), Buchtová et al. (12–14), Tiwary et al. (15), and Tolarová et al. (16)], as well as in haematological or biochemical profiles (2, 10, 15–20), immune performance (2, 10, 16, 21), and behavior (15, 22).

Host–parasite interactions are often characterized by narrow genetic specificity (23), which may explain why parasites can reflect changes in host ploidy status. Higher allelic diversity at immune-related genes in polyploid hosts may allow recognition of a broader range of parasite species (24). Moreover, if additional genome copies are expressed, polyploids may produce greater quantities of immune-related gene products (3), making it more difficult for parasites to evade host recognition mechanisms (24, 25). However, polyploidy may not necessarily generate functional diversity unless increased allelic variation is reflected at the protein level.

The impacts of polyploidy on immune defense in fish have been documented [e.g., Šimková et al. (21), Hakoyama et al. (26), Langston et al. (27), and Fuad et al. (28)]. For example, triploid gynogenetic females of goldfish (*Carassius auratus* L., 1758) exhibited significantly higher parasite load than diploid sexual females, which was explained by reduced phagocytic activity in triploid form (26). In the study by Šimková et al. (21), the most common triploid MHC (major histocompatibility complex) genotype of gibel carp (*Carassius gibelio* Bloch, 1782) was more heavily parasitized by gill monogeneans compared to diploids and less frequent triploid MHC variants from the same population. Fuad et al. (28) reported high infection levels of eye trematodes, and underexpression of numerous immunity-related genes in gynogenetic triploid gibel carp compared to sexual diploids, supporting the idea that certain loci, or even whole genomes, may be up- or down-regulated (or even silenced) as ploidy increases, a phenomenon documented in allopolyploids (3). However, polyploidy may produce non-uniform effects on parasites, as demonstrated in plants and phytophagous moths (29), showing that polyploid plants less infected by one phytophagous insect species were significantly more infected by two others.

The present study aimed to investigate the effect of host ploidy status on metazoan parasite infection in tench (*Tinca tinca* L., 1758, Cyprinidae), a commercially important fish species widely bred in European pond aquaculture. The Czech Republic is one of the leading producers of marketable tench in the Europe. Its production relies either on natural spawning of selected broodstock under controlled conditions or completely on artificial reproduction (30). Artificial production of triploid tench has been shown to result in higher marketable weight and improved product quality (11). However, a high incidence of spontaneous triploidy has also been observed in farmed tench, attributed to natural predisposition for egg overripening and the presence of a recessive allele responsible for the failure of the second polar body extrusion when homozygous (31).

Growth, physiological traits, and immune response of diploid and triploid tench have been extensively studied (11, 16, 17), and the health status of farmed tench has been previously investigated by Svobodová and Kolářová (32) and Piačková and Flajšhans (33). In this integrative study of tench health, we focused on year-long temporal variation in metazoan parasite load, as well as condition, haematological, biochemical, and immunological parameters, to assess (1) differences in parasite susceptibility between induced triploid and amphimictic diploid tench and (2) the potential effect of metazoan parasite infection on fish condition, physiology, and health status.

## 2 Materials and methods

### 2.1 Host and parasite collection

Farmed amphimictic diploid and induced triploid specimens of tench were sampled from a breeding pond located at the South Bohemian Research Centre of Aquaculture and Biodiversity of Hydrocenoses in Vodňany, Czech Republic. Triploid tench was produced by cold shock as described by Flajšhans et al. (34). Fish sampling was conducted using seine netting during four seasonal periods (water temperature was measured at the time of fish sample collection): September (16.9 °C), December (2.1 °C), March (7.4 °C), and June (16.4 °C). A total of 160 tench aged five to six years were examined, including 81 diploid and 79 triploid specimens.

Immediately after capture, skin mucus was gently stripped and placed into sterile tube. Blood samples were drawn from the caudal vein following Pravda and Svobodová (35) and mixed with heparin (50 IU.ml^−1^ of blood, Zentiva). Fish were subsequently transported to the laboratory and individually euthanised by spinal transection prior to dissection. Standard and total body length (in mm), total body weight, and eviscerated (without internal organs) body weight (to the nearest 0.1 g) were recorded. Wet weights of gonads, liver and spleen were measured (to the nearest 0.001 g) and used for calculation of physiological indices: hepatosomatic index (HSI), splenosomatic index (SSI), and gonadosomatic index (GSI) [see Rohlenová et al. (36) for more details]. The number of fish examined per group (ploidy/sampling period), total length, and all physiological parameters are presented in Table 1. Ploidy level of each specimen was verified from the blood sample by means of flow cytometry as relative DNA content in peripheral blood cells. Samples were processed according to Vindelov and Christensen (37).

**Table 1:** Sample size (N), total fish length (in mm, mean±SD), parasite load, physiological, biochemical and immunological parameters (mean±SD is shown for each parameter) for amphimictic diploid (2n) and induced triploid (3n) tench.

Fish parameters	September		December		March		June	
	2n	3n	2n	3n	2n	3n	2n	3n
N	18	22	20	20	22	18	21	19
Total length	227 ± 18	240 ± 24	228 ± 21	246 ± 26	237 ± 21	251 ± 32	243 ± 27	256 ± 29
Parasite load								
Prevalence	39%	46%	100%	100%	100%	89%	91%	95%
Abundance	20.0 ± 39.9	2.1 ± 4.4	24.8 ± 22.0	18.6 ± 13.5	27.1 ± 36.8	12.0 ± 14.4	389.5 ± 405.7	124.5 ± 144.4
Intensity of infection	51.4 (1–124)	4.5 (1–21)	24.8 (1–78)	18.6 (2–48)	27.1 (1–156)	13.5 (2–57)	430.5 (8–1746)	131.4 (1–446)
Species richness	4	6	3	4	8	7	6	6
Physiological parameters								
Fultonʼs condition factor	1.98 ± 0.15	2.01 ± 0.12	2.14 ± 0.20	2.10 ± 0.16	2.03 ± 0.13	2.03 ± 0.12	2.08 ± 0.20	2.05 ± 0.19
HSI	2.53 ± 0.58	2.71 ± 0.55	3.24 ± 0.75	3.45 ± 0.58	2.43 ± 0.46	2.44 ± 0.54	1.83 ± 0.31	1.86 ± 0.38
SSI	0.39 ± 0.14	0.36 ± 0.16	0.44 ± 0.18	0.41 ± 0.11	0.34 ± 0.14	0.37 ± 0.07	0.38 ± 0.14	0.45 ± 0.13
GSI - males	0.22 ± 0.07	0.16 ± 0.05	0.69 ± 0.21	0.47 ± 0.17	0.77 ± 1.31	0.39 ± 0.24	0.98 ± 0.20	0.68 ± 0.22
GSI - females	3.13 ± 1.63	1.58 ± 0.69	3.49 ± 0.68	2.15 ± 0.62	2.83 ± 0.81	1.76 ± 0.70	16.65 ± 5.41	6.06 ± 4.94
Biochemical parameters								
Glucose	9.1 ± 2.1	8.4 ± 3.0	9.7 ± 1.9	11.3 ± 4.0	6.8 ± 1.3	6.5 ± 0.9	5.0 ± 1.1	5.8 ± 1.7
11-ketotestosterone	810 ± 832	548 ± 341	218 ± 87	150 ± 70	6,494 ± 3,282	8,828 ± 2,499	7,718 ± 6,707	6,308 ± 7,308
Haematological parameters								
Erythrocyte count	1.84 ± 0.30	1.23 ± 0.22	1.67 ± 0.26	1.02 ± 0.20	1.78 ± 0.34	1.01 ± 0.24	1.73 ± 0.32	1.12 ± 0.19
Haemoglobin	99.3 ± 16.5	86.8 ± 13.7	91.5 ± 12.2	79.1 ± 9.3	92.8 ± 15.0	55.3 ± 8.1	88.4 ± 12.6	80.3 ± 8.9
Haematocrit	0.36 ± 0.06	0.32 ± 0.05	0.30 ± 0.04	0.26 ± 0.03	0.32 ± 0.04	0.26 ± 0.04	0.31 ± 0.07	0.30 ± 0.03
Leukocrit	0.016 ± 0.007	0.015 ± 0.006	0.010 ± 0.003	0.011 ± 0.006	0.011 ± 0.004	0.021 ± 0.005	0.020 ± 0.009	0.024 ± 0.013
Immunological parameters								
Leukocyte count	95.9 ± 51.9	76.3 ± 34.3	38.8 ± 25.2	27.5 ± 15.5	92.7 ± 33.5	60.2 ± 19.8	121.6 ± 84.2	93.1 ± 46.4
Respiratory burst (peak)	132 ± 128	176 ± 143	34 ± 42	58 ± 47	34 ± 30	86 ± 60	70 ± 62	97 ± 50
Complement activity	2.65 ± 0.49	2.12 ± 0.46	2.17 ± 1.01	2.23 ± 0.78	1.75 ± 0.63	1.98 ± 0.49	1.39 ± 0.73	1.83 ± 0.95
Lysozyme concentration	0.39 ± 0.48	0.39 ± 0.57	0.01 ± 0.03	0.01 ± 0.02	0.24 ± 0.27	0.10 ± 0.15	0.05 ± 0.07	0.04 ± 0.05

HSI, hepatosomatic index; SSI, splenosomatic index; GSI, gonadosomatic index. Mean and range (minimum-maximum) is shown for parasite intensity of infection. Mean±SD is shown for parasite abundance.

Tench specimens were screened for metazoan parasites using binocular stereomicroscopes. All parasites were removed from examined organs and preserved according to standard parasitological procedures (38). Monogenea and Myxosporea were preserved as semi-permanent slides using a mixture of ammonium picrate and glycerine (39). Digenea, Cestoda, Mollusca, Crustacea, and Acarina were preserved in 4% formaldehyde or 70% ethanol (38), with digeneans and cestodes subsequently stained with ferric acetocarmine and mounted in Canada balsam (40). Parasites were identified using a light microscope (Olympus BX 50) equipped with phase-contrast, differential interference contrast (DIC according to Nomarski), and measured using Olympus Digital Image Analysis.

Characteristics of parasite infection, including prevalence, abundance, and intensity of infection, were calculated according to Bush et al. (41). Prevalence was defined as the percentage of fish infected by a given parasite species in a sample; mean abundance as the average number of parasites per host (including infected and uninfected individuals); and mean intensity of infection as the average number of parasites per infected host. Intensity range indicates the minimum and maximum number of parasites per infected host. Parasite species richness, defined as the total number of parasite species, was calculated for each fish group and per individual host. Parasite species strictly associated with tench were classified as specialists [e.g., Gusev (42) and Našincová and Scholz (43)], while those with a broad host range [based on published studies, e.g., Moravec (44)] were considered generalists (Table 2).

**Table 2:** Prevalence (P in %) and mean abundance and maximum intensity of infection (A, I) of metazoan parasites infecting farmed amphimictic diploid (2n) and induced triploid (3n) tench.

Parasite taxa		September		December		March		June	
		2n	3n	2n	3n	2n	3n	2n	3n
		P (A, I)	P (A, I)	P (A, I)	P (A, I)	P (A, I)	P (A, I)	P (A, I)	P (A, I)
Myxosporea									
Myxosporea spp.	-	6 (0.33, 6)	9 (0.09, 1)	55 (8.35, 69)	45 (6.40, 32)	18 (2.23, 16)	6 (0.11, 2)		11 (0.11, 1)
Monogenea									
*Gyrodactylus tincae*	S		9 (0.09, 1)	95 (10.10, 46)	95 (7.90, 35)	86 (8.50, 45)	89 (10.11, 57)	43 (0.57, 2)	47 (0.74, 3)
Digenea									
*Asymphylodora tincae*	S	33 (19.5, 124)	5 (0.73, 16)	40 (6.30, 61)	25 (3.35, 28)	32 (14.82, 149)	6 (0.11, 2)	90 (388.19, 1744)	74 (119.37, 440)
*Diplostomum* spp.	G							10 (0.19, 3)	
*Tylodelphys clavata*	G		5 (0.18, 4)			5 (0.09, 2)		10 (0.14, 2)	
Cestoda									
*Caryophyllaeus* spp.	G	6 (0.06, 1)				18 (1.36, 22)	11 (0.94, 10)		5 (2.11, 40)
*Khawia baltica*	G		5 (0.05, 1)						
*Neogryporhynchus cheilancristrotus*	G					5 (0.05, 1)			
*Valipora campylancristrota*	G	6 (0.11, 2)	36 (0.91, 5)		30 (0.90, 8)	5 (0.05, 1)	22 (0.22, 1)	19 (0.24, 2)	58 (1.53, 5)
Mollusca									
*Anodonta* sp.	G					5 (0.05, 1)			
Crustacea									
*Argulus foliaceus*	G							19 (0.19, 1)	42 (0.63, 3)
*Ergasilus sieboldi*	G						11 (0.17, 2)		
Acarina									
*Hydrozetes* sp.	G						11 (0.33, 5)		

Host specificity of parasites is shown – specialists (S) and generalists (G). Dash indicates undetermined specificity.

### 2.2 Haematological, biochemical, and immunological parameters

Total erythrocyte count (expressed in T.l^−1^) and leukocyte count (in G.l^*−*1^) were determined using a Bürker hemocytometer after dilution of heparinised blood in Natt-Herrick solution at 1:200 ratio (45, 46). Haemoglobin content (in g.l ^−1^) was assessed photometrically at 540 nm (Helios Unicam, USA), using Kampen-Zijlster transformation medium (33). Haematocrit and leukocrit values (in l.l^*−*1^) were measured using 75 mm long heparinized microcapillaries with an inner volume of 60 μm. Centrifugation was performed at 12,000 g for 3 min using a haematocrit centrifuge (45).

Blood samples for respiratory burst activity were prepared for each fish according to Buchtíková et al. (47) and analyzed within two hours of collection. The kinetics of luminol-enhanced chemiluminescence were measured at 22 °C for one hour at room temperature using LM01-T luminometer (Immunotech, Czech Republic). The peak curve, expressed in relative light units (RLU) represented the maximal intensity of respiratory burst. Complement activity was assessed following Buchtíková et al. (47) and calculated as the inverse of the time (in h^−1^) required to kill 50% of *Escherichia coli*, based on the difference between the maximum measurement time (4 h) and the observed time to 50% lysis.

Lysozyme concentration in skin mucus (in mg.ml^−1^) was determined by radial diffusion in agarose containing *Micrococcus luteus* (CCM 169) according to Poisot et al. (48). The concentration of 11-ketotestosterone (in pg.ml^−1^) in male plasma was measured using a commercial competitive enzyme immunoassay kit (Cayman Chemical, Estonia). Plasma glucose levels (in mmol.l^−1^) were analyzed using the commercial Biolatest Glukosa Liquid 500 kit (PLIVA-Lachema Diagnostika s.r.o., Brno, Czech Republic). All measured haematological, biochemical, and immunological parameters are presented in Table 1.

### 2.3 Data analysis

The effects of season, ploidy, and sex on response variables were tested using either linear models (LM) or generalized linear models (GLM). GLMs were applied to all parasitological characteristics and leukocyte count (all assumed to follow a negative binomial distribution, except parasite species richness, which was modelled using a quasi-Poisson distribution). LMs were used for all other condition, physiological, and immunological parameters.

The null model for each response variable included season, ploidy, and sex as predictors. Interactions were excluded due to limited number of replications. For GSI (analyzed separately for males and females) and 11-ketotestosterone (measured only in males), the null model contained only season and ploidy. A backward stepwise approach was used to simplify models by removing predictors that did not significantly affect the fit of the model, as determined by likelihood ratio tests (49). Tukey’s HSD test was applied to control for Type II error in multiple *post-hoc* pairwise comparisons [using the glht and mcp functions from *multcomp* package; Hothorn et al. (50)]. Where necessary, i.e., when visual inspection of residuals revealed patterns indicating biased models, such as trends in residuals and their variance, the response variables were log-transformed using log (x + 1), and the model was re-evaluated. This adjustment was applied to male and female GSI, 11-ketotestosterone, leukocrit, lysozyme concentration, respiratory burst peak, and glucose level.

Spearman correlation tests were used to assess associations between total parasite abundance or abundance of the four most abundant parasite species and fish condition, physiological, haematological, and immunological parameters, calculated separately for diploids and triploids. Bonferroni correction was applied to adjust significance levels and reduce the probability of committing a Type I error. All statistical analyses were performed in R version 4.1.2 (51), using the *MASS* (52), *multcomp* (50), and *emmeans* (53) packages.

## 3 Results

### 3.1 Parasite load

**Figure 1:**
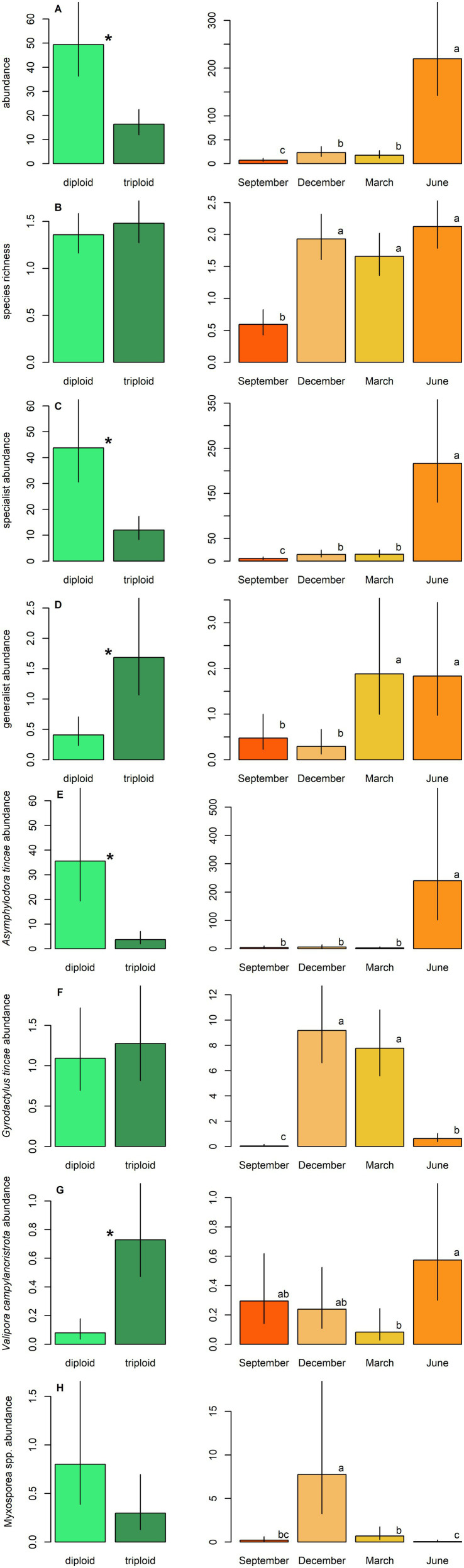
Parasite abundance and species richness in tench, differences between diploids and triploids (green bars) and among sampled seasons (orange bars) are shown. **(A)** Total abundance, **(B)** species richness, **(C)** abundance of specialists, **(D)** abundance of generalists, **(E)** abundance of *Asymphylodora tincae*, **(F)** abundance of *Gyrodactylus tincae*, **(G)** abundance of *Valipora campylancristrota*, **(H)** abundance of Myxosporea spp. Ploidy bars with *differ significantly from each other. Season bars with the different letters differ significantly from each other.

A total of thirteen taxa of parasitic metazoans were identified on farmed tench, with ten taxa present in diploid and ten in triploid fish (Table 2). Parasite species richness did not differ significantly between ploidy levels (GLM, *p* > 0.05; Figure 1B). However, the overall parasite abundance was significantly lower in triploids compared to diploids (GLM, *p* < 0.001; Figure 1A). Triploids exhibited significantly reduced abundance of specialist parasites (GLM, *p* < 0.001), whereas the abundance of generalists was significantly lower in diploids than in triploids (GLM, *p* < 0.001) (Table 3; Figures 1C,D).

**Table 3:** Contribution of three predictors (season, ploidy and sex) in models explaining tench parasite assemblage characteristics.

Response variable	Ploidy	Season	Sex	Dfn
Species richness	0.452	**<0.001**	0.409	159
Abundance	**<0.001**	**<0.001**	0.283	159
Specialist abundance	**<0.001**	**<0.001**	0.294	159
Generalist abundance	**<0.001**	**<0.001**	0.149	159
*Asymphylodora tincae*	**<0.001**	**<0.001**	0.949	159
*Gyrodactylus tincae*	0.472	**<0.001**	**<0.001**	159
*Valipora campylancristrota*	**<0.001**	**0.019**	0.528	159
Myxosporea spp.	0.153	**<0.001**	0.388	159

For each predictor, *p*-value of likelihood-ratio tests comparing the model with and without the respective predictor is shown. *p*-values of final model parameters (*p* < 0.05) are in bold. Dfn - degrees of freedom for null model (df season = 3, ploidy = 1 and sex = 1).

Four taxa were consistently present throughout the year: the monogenean *Gyrodactylus tincae* Malmberg, 1957, the adult trematode *Asymphylodora tincae* (Modeer, 1790), the larval cestode *Valipora campylancristrota* (Wedl, 1855), and an unspecified group of myxosporeans (Myxosporea spp.) (Table 2). *Asymphylodora tincae* was the most abundant and one of two the most prevalent parasite species, reaching also the highest maximum intensity of infection, followed by *G. tincae* as the second most abundant. Higher abundance of *A. tincae* along with lower abundance of *V. campylancristrota* was observed in diploid tench (GLM, both *p* < 0.001) (Table 3; Figures 1E,G). No significant effect of ploidy was observed on the abundance of Myxosporea spp. or *G. tincae* (GLM, both *p* > 0.05) (Table 3; Figures 1F,H).

Sex had no significant effect on most parasitological parameters, with the exception of abundance of *G. tincae*, which was significantly lower in males than in females (GLM; *p* < 0.001; Table 3). Seasonal variation significantly affected all measured characteristics of parasite infection (GLM; *P* all < 0.05; Table 3; Figure 1). Overall parasite abundance, as well as abundance of *A. tincae*, *V. campylancristrota*, and specialist parasites (see Table 2), peaked in June (Figures 1A,C,E,G). Generalist abundance was highest in March and June (Figure 1D), while the Myxosporea spp. and *G. tincae* reached their highest abundance in December or December and March, respectively (Table 3; Figures 1F,H). Overall, parasite infection levels were lowest in September.

### 3.2 Physiological parameters

**Figure 2:**
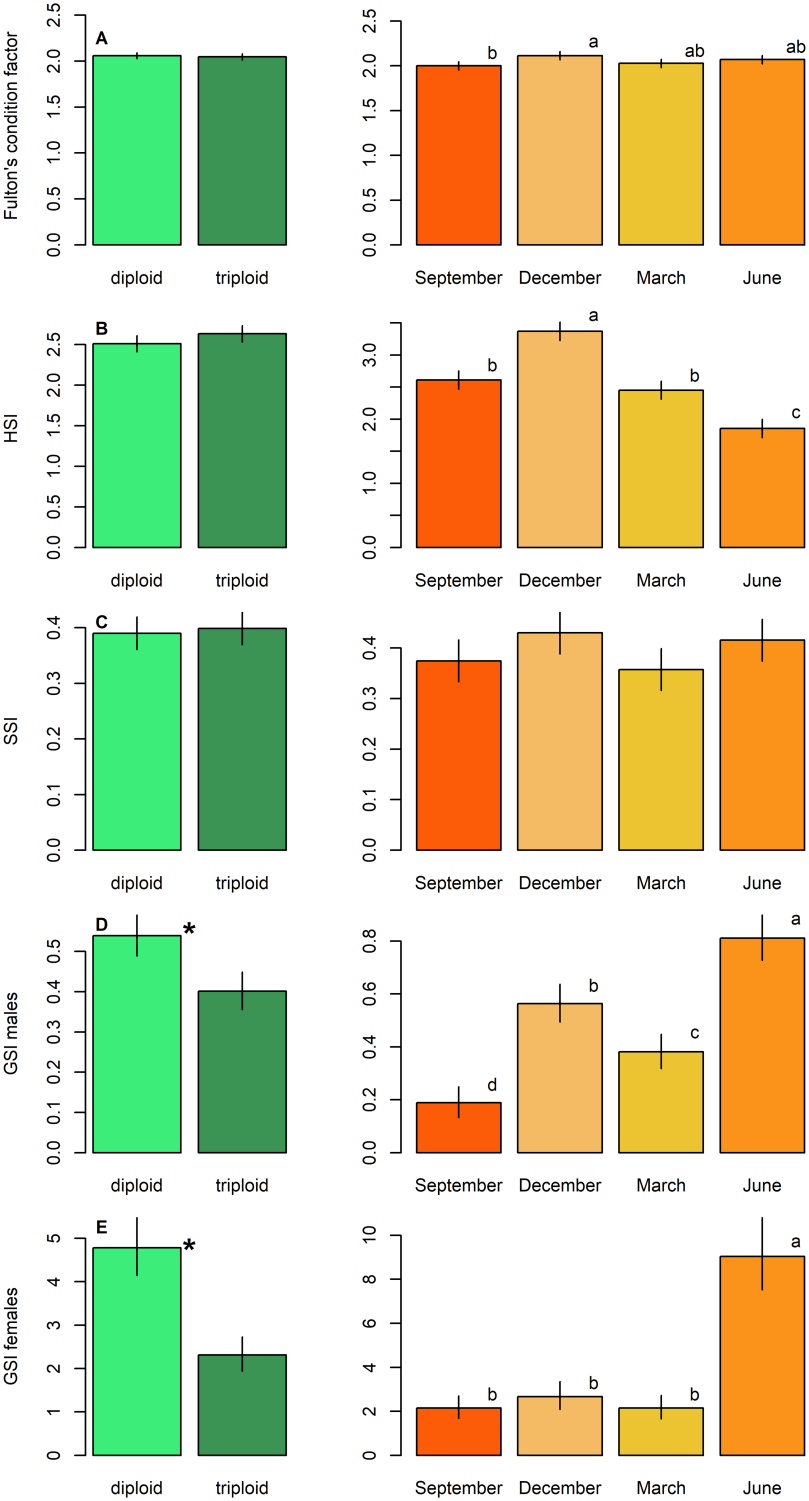
Physiological parameters, including: **(A)** Fulton’s condition factor, **(B)** hepatosomatic index (HSI), **(C)** splenosomatic index (SSI), and **(D)** gonadosomatic index in males (GSI males) and **(E)** females (GSI females) in diploid and triploid tench (green bars), and their seasonal fluctuation (orange bars). Ploidy bars with *differ significantly from each other. Season bars with the different letters differ significantly from each other.

GSI was significantly higher in diploids compared to triploids for both sexes (LM, *p* < 0.001; Table 4; Figures 2D,E). Other condition-related parameters showed no significant dependence on ploidy (LM, *p* > 0.05, Table 4). Seasonal variation significantly affected all condition parameters (Table 4; Figure 2). GSI peaked in June for both sexes, while HSI reached its highest in December and lowest in June. Fulton’s condition coefficient remained relatively stable across seasons, with December values significantly exceeding those recorded in September (Figure 2A). SSI and HSI were higher in females than in males, whereas the opposite trend was observed in Fulton’s condition coefficient (Table 4).

**Table 4:** Contribution of three predictors (season, ploidy and sex) in models explaining tench physiological characteristics.

Response variable	Ploidy	Season	Sex	Dfn
GSI males	**<0.001**	**<0.001**		81
GSI females	**<0.001**	**<0.001**		77
HSI	0.086	**<0.001**	**<0.001**	159
SSI	0.672	**0.002**	**0.048**	159
Fultonʼs condition factor	0.550	**0.004**	**<0.004**	159

For each predictor, *p*-value of likelihood-ratio tests comparing the model with and without the respective predictor is shown. *p*-values of final model parameters (*p* < 0.05) are in bold. Dfn - degrees of freedom for null model (df season = 3, ploidy = 1 and sex = 1).

### 3.3 Haematological, biochemical and immunological parameters

**Figure 3:**
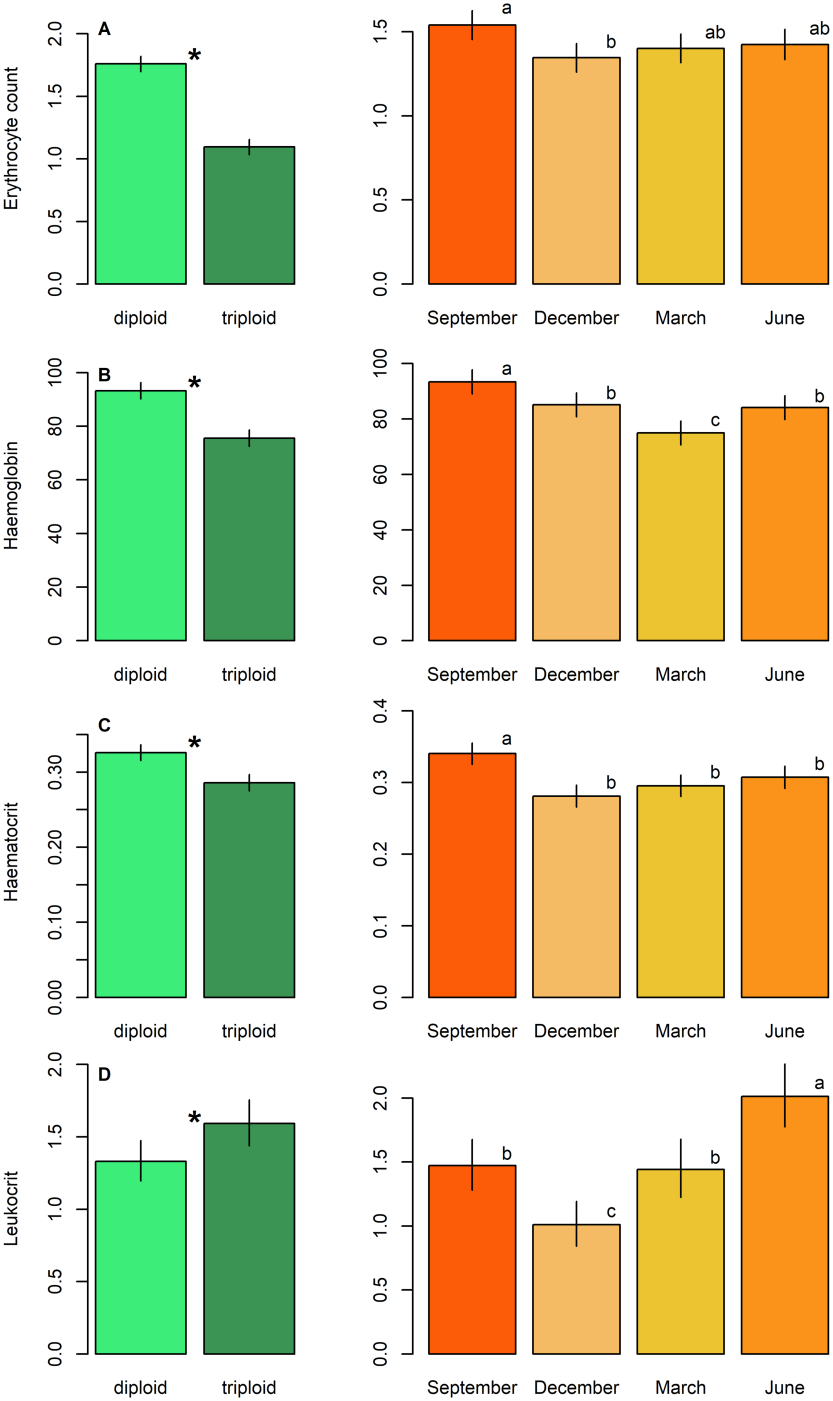
Haematological parameters, including: **(A)** Erythrocyte count, **(B)** haemoglobin concentration, **(C)** haematocrit and **(D)** leukocrit in plasma samples of diploid and triploid tench (green bars), and their seasonal fluctuation (orange bars). Ploidy bars with * differ significantly from each other. Season bars with the different letters differ significantly from each other.

**Figure 4:**
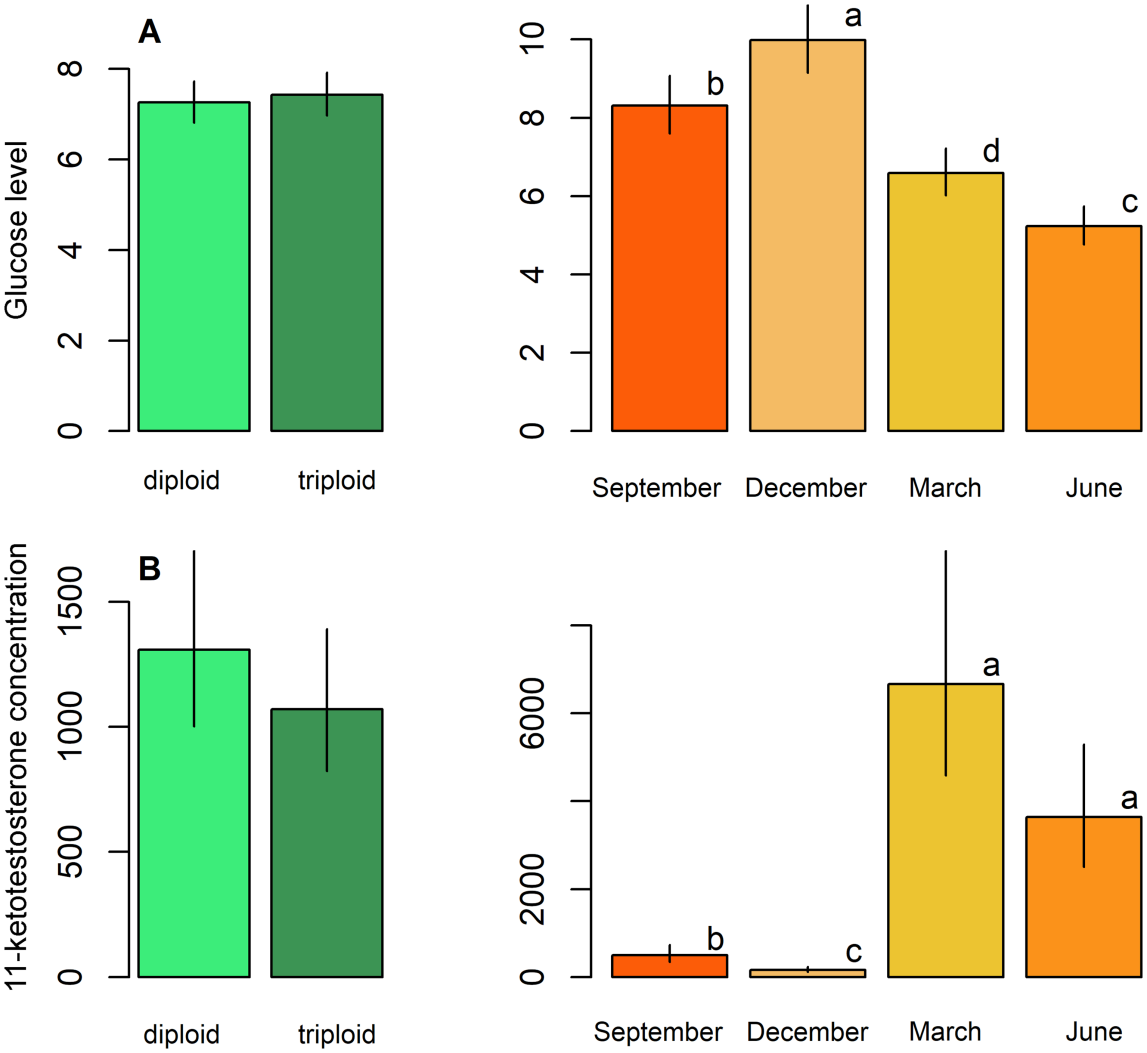
Biochemical parameters, including: **(A)** Glucose level and **(B)** 11-ketotestosterone concentration in males in plasma samples of diploid and triploid tench (green bars), and their seasonal fluctuation (orange bars). Season bars with the different letters differ significantly from each other.

**Figure 5:**
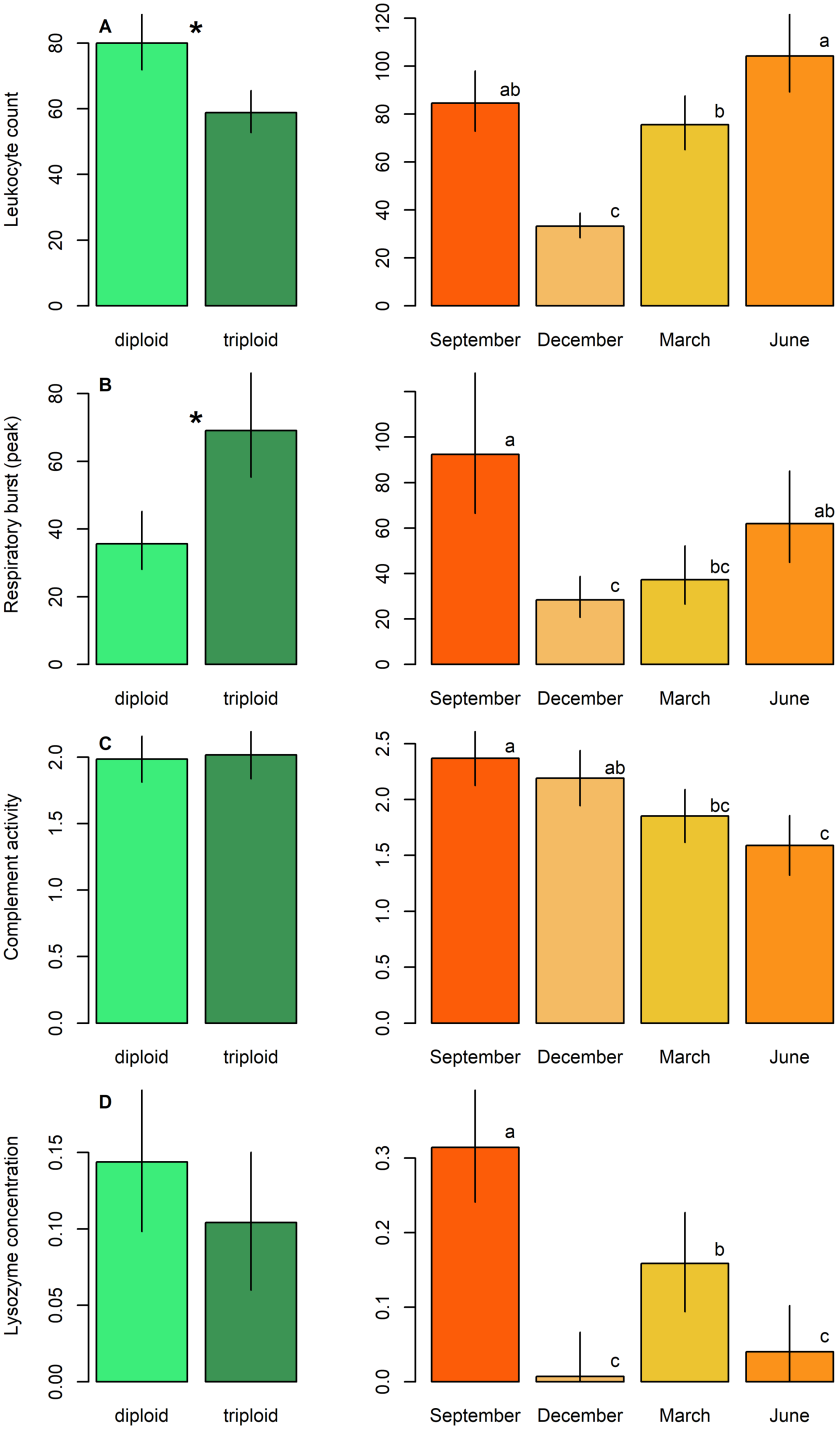
Immunological parameters, including: **(A)** Leukocyte count, **(B)** respiratory burst peak, **(C)** complement activity, and **(D)** lysozyme concentration in diploid and triploid tench (green bars), and their seasonal fluctuation (orange bars). Ploidy bars with *differ significantly from each other. Season bars with the different letters differ significantly from each other.

Diploids exhibited higher erythrocyte counts, haematocrit, and haemoglobin content compared to triploids, while the reverse pattern was observed for leukocrit (Table 5; Figure 3). No significant effect of ploidy was found for glucose levels and 11-ketotestosterone concentrations (Table 5; Figure 4). Leukocyte counts were significantly higher in diploids (Figure 5A), whereas triploids showed elevated respiratory burst peak (Figure 5B). No ploidy-related differences were detected in complement activity or lysozyme concentration (Table 5; Figures 5C,D). Haematocrit, leukocrit, and leukocyte counts were significantly higher in females than in males (Table 5).

**Table 5:** Contribution of three predictors (season, ploidy and sex) in models explaining tench haematological, biochemical, and immunological parameters.

Response variable	Ploidy	Season	Sex	Dfn
Erythrocyte count	**<0.001**	**0.014**	0.455	154
Heamoglobin	**<0.001**	**<0.001**	0.109	159
Haematocrit	**<0.001**	**<0.001**	**0.038**	156
Leukocrit	**0.013**	**<0.001**	**0.012**	137
Glucose level	0.598	**<0.001**	0.881	159
11-ketotestosteron	0.272	**<0.001**		80
Leukocyte count	**<0.001**	**<0.001**	**0.003**	155
Respiratory burst	**<0.001**	**<0.001**	0.248	145
Complement activity	0.799	**<0.001**	0.442	142
Lysozyme concentration	0.219	**<0.001**	0.854	158

For each predictor, *p*-value of likelihood-ratio tests comparing the model with and without the respective predictor is shown. *p*-values of final model parameters (*p* < 0.05) are in bold. Dfn - degrees of freedom for null model (df season = 3, ploidy = 1 and sex = 1).

Seasonal variation was evident across all measured parameters. Erythrocyte count, haematocrit, and haemoglobin peaked in September, while leukocrit was highest in June and lowest in December (Figure 3). Glucose levels peaked in December, and 11-ketotestosterone concentrations were highest in March and June (Table 5; Figure 4). Respiratory burst peak, lysozyme concentration, and complement activity reached their maximum in September (Figures 5B–D). The lowest values of respiratory burst peak and leukocyte count were observed in December, while leukocyte counts peaked in June (Table 5; Figures 5A,B).

### 3.4 The associations between parasite load and host parameters

Total parasite abundance was positively associated with GSI in both sexes, regardless of ploidy. However, this association became insignificant in triploid females following Bonferroni correction (Table 6). In diploids, parasite abundance was negatively associated with glucose levels and lysozyme concentration (*r* = −0.437 and −0.380, respectively; both *p* < 0.001). Other physiological, biochemical, haematological, and immunological parameters showed no consistent associations with parasite abundance, or their significance diminished after correction for multiple analyses - specifically HSI, haemoglobin, and complement activity in diploids, and lysozyme concentration in triploids (Table 6).

**Table 6:** Spearman correlation coefficients (r) and their respective *p*-values for correlations between total parasite abundance and fish physiological (Fulton’s condition factor, hepatosomatic index (HSI), splenosomatic index (SSI), gonadosomatic index (GSI) in males and females); haematological (erythrocyte count, haemoglobin content, haematocrit, leukocrit); biochemical (glucose level in plasma, 11-ketotestosterone concentration in males); and immunological (leukocyte count, respiratory burst peak, complement activity, lysozyme concentration) parameters, calculated separately for diploids (2n) and triploids (3n).

Fish parameters	*r*		*p*	
	2n	3n	2n	3n
Fulton’s condition	−0.009	0.076	0.939	0.503
HSI	***−0.307***	−0.109	***0.005***	0.341
SSI	−0.025	0.072	0.827	0.527
GSI-males	**0.560**	**0.537**	**<0.001**	**<0.001**
GSI-females	**0.648**	***0.354***	**<0.001**	***0.029***
Erythrocyte count	−0.127	−0.037	0.267	0.751
Haemoglobin	***−0.320***	−0.100	***0.004***	0.380
Haematocrit	−0.203	−0.172	0.071	0.134
Leukocrit	0.156	0.109	0.197	0.375
Glucose	**−0.437**	−0.068	**<0.001**	0.549
11-ketotestosterone	0.226	0.112	0.161	0.484
Leukocyte count	0.113	−0.073	0.321	0.528
Respiratory burst	0.011	−0.127	0.929	0.269
Complement	***−0.253***	0.120	***0.030***	0.328
Lysozyme	**−0.380**	***−0.247***	**0.001**	***0.028***

All significant correlations (*p* < 0.05) are in bold. Non-significant correlations after Boferroni correction (0.0033 > *p* < 0.05) are in bold italics. HSI - 2n, GSI females - 3n, haemoglobin - 2n, complement - 2n, and lysozyme - 3n.

The abundance of *A. tincae* tended to increase with GSI in both sexes and decrease with HSI and glucose levels in diploids. Abundance of *G. tincae* tended to increase with HSI in both sexes, while negative associations were found with haematocrit and haemoglobin in triploids, and with leukocrit and respiratory burst peak in diploids, after Bonferroni corrections. No significant associations were found between abundance of *V. campylancristrota* and host parameters following correction. In contrast, abundance of Myxosporea spp. (present on the gills) was negatively correlated with leukocyte counts in both diploid a triploid tench (Supplementary Table S1).

## 4 Discussion

Investigating parasite load in diploid and triploid individuals of commercially important fish is a crucial aspect of assessing fish health in aquaculture. Artificially induced polyploid fish are of particular interest due to their enhanced growth performance, improved organoleptic qualities, and greater economic viability across diverse farming environments (10). Polyploidization is associated with changes in cell morphology and physiology, which can affect fish fitness and resistance to pathogens (2, 3, 54). Although polyploids are expected to exhibit advantages in coping with environmental stressors, including pathogens, empirical evidence remains inconclusive [e.g., Osnas and Lively (25), Hakoyama et al. (26), Nuismer and Thompson (29), Ozerov et al. (55), and Van de Peer et al. (56)].

In this study, we examined the relationship between ploidy level and metazoan parasite infection in tench over a one-year period, encompassing four seasonal sampling points: autumn, winter, early spring, and summer. Our findings indicate that ploidy status plays an important role in parasite infection in tench aquaculture. Induced triploids tended to have lower parasitic load than amphimictic diploids over the course of the year, primarily due to higher infection rates of diploids by the most abundant host-specific digenean parasite *A. tincae*, which peaked in summer. Adult *A. tincae* are found exclusively in digestive tract of tench; previous reports of its occurrence in other fish species likely stem from misidentification or confusion with other species of the genus *Parasymphylodora* that infect different fish hosts (43, 57). Tench serve as definitive hosts, becoming infected through ingestion of pulmonate snails carrying rediae with mature cercariae (43).

Conversely, triploid tench reached higher prevalence of generalist endoparasite *V. campylancristrota* across all seasons, with a summer peak. However, the abundance remained low throughout the year. This cestode parasitizes a broad range of freshwater fish, which act as second intermediate hosts. Infection occurs via ingestion of cyclopoid copepods, with the plerocercus stage residing in the fish gall bladder until consumed by the definitive bird host (58).

The contrasting infection patterns of *A. tincae* and *V. campylancristrota* between diploid and triploid tench suggest that polyploidy may exert non-uniform effects on host–parasite interactions. A similar phenomenon was observed by Nuismer and Thompson (29) in *Heuchera grossulariifolia* Rydb., where polyploid plants were less attacked by one phytophagous insect species but more susceptible to others.

Several mechanisms may explain the observed difference in infection by the two most common parasite species. Increased ploidy may not uniformly enhance resistance; it can either strengthen or weaken host defenses depending on allele interactions between hosts and parasites. Triploid fish may keep three distinct alleles per single locus, providing them with higher levels of heterozygosity what can potentially accrue associated fitness benefits over diploid fish (1). Greater heterozygosity may impede parasite evasion of host recognition, potentially conferring increased resistance (59). Thus, we can hypothesize that the reduced susceptibility of triploid tench to *A. tincae* infection may be linked to their higher allelic diversity and expression of immune-related genes, although these aspects were not directly assessed in this study. To the best of our knowledge, no any study yet dealt directly with the assessment of heterozygosity in triploid tench but several studies on farmed crustacean, shellfish and fish species revealed higher heterozygosity levels for induced triploids if compared to their diploid counterparts, with the magnitude and/or detectability of triploidy-induced changes in heterozygosity depending greatly on the genetic background of the population studied, as recently reviewed, e.g., by Flajšhans et al. (60). Furthermore, host–parasite interactions often involve tight genetic specificity, which may favour polyploid advantages against coevolving parasites (25), such as host-specific species like *A. tincae.* Beyond genetic factors, host specificity of parasites is also shaped by the ecological, behavioral, physiological, and biochemical traits of hosts (23).

Physiological and feeding behavioral differences between diploid and triploid tench may further contribute to variation in parasite abundance. Triploid organisms are generally characterized by a reduced number of cells that are proportionally larger in size (61), a phenomenon well documented also in fish species (62). Reduction in cell numbers in central nervous system and reduced hormone levels of hormones in triploid fish could result in diminished responsiveness to environmental stimuli leading to expression of altered behavior (2, 15). Triploids have also been reported to be less aggressive than diploids, potentially affecting competitive feeding success (22, 63). In our study, both ploidy groups were reared in the same pond under conditions of potential food competition.

The other two most abundant parasite taxa were the ectoparasitic host-specific monogenean *G. tincae* and undetermined Myxosporea spp. *Gyrodactylus* parasites, due to their hyperviviparity and rapid reproduction (a single worm can produce thousands of progeny), may pose a significant threat in aquaculture (64–66). In polyploid fish, triploid Atlantic salmon were found to be more susceptible to *Gyrodactylus salaris* Malmberg, 1957, probably due to impaired complement-dependent immune responses (55). In our study, unlike *A. tincae* and *V. campylancristrota*, the prevalence and abundance of *G. tincae* and Myxosporea spp. peaked in winter and early spring (or winter only), with no significant differences between diploid and triploid tench. Notably, the maximum intensity of infection of the host-specific *A. tincae* was 30 times higher than that of *G. tincae*. Piačková and Flajšhans (33) reported a higher prevalence of undetermined *Gyrodactylus* spp. in meiotic gynogenic tench, with no apparent difference between induced triploids and amphimictic diploids, consistent with our findings. However, their conclusions were based solely on visual estimates of parasite prevalence as their study did not include data on intensity of infection. In contrast, our study supports this observation by providing original quantitative data of metazoan parasite load.

Previous studies on polyploid fish [e.g., Benfey (2), Ballarin et al. (18), Peruzzi et al. (19), Levy-Pereira et al. (67), and Rożyński et al. (68)], including those focussing on tench (11, 16, 17, 69), have highlighted that ploidy can alter fish condition, physiology, haematological profile, and immune function. Although induced triploid tench generally exhibit faster growth and improved weight gain [as reviewed by Flajšhans et al. (11)], our study did not reveal differences in body condition factor, energy reserves (measured by HSI), or immunocompetence (expressed by SSI). However, reproductive investment (measured by GSI) followed the trend described in previous tench studies, with diploid females and males showing higher GSI values compared to their triploid counterparts (12, 70). Regarding reproductive potential, triploid females are sterile, while triploid males may produce aneuploid spermatozoa with variable DNA content capable of initiating embryo development (11). Histological studies have revealed underdeveloped or retarded ovaries in females and testes in males (13, 70).

Haematological parameters of 6 + year-old induced triploid and amphimictic diploid tench were previously analyzed by Tolarová et al. (16), who reported lower erythrocyte counts (compensated by larger erythrocyte size), haemoglobin, and haematocrit in triploids compared to diploids, suggesting reduced oxygenation capacity and potentially less effective immune response (11). Similar findings have been reported in other studies on farmed tench (17, 33). A comparable pattern was observed for leukocyte counts in our study and has also been previously documented in various triploid fish (2, 19, 69, 71).

However, we found an opposite trend for leukocrit, with significantly higher values in triploids than in diploids. Since leukocrit reflects the volume fraction of white blood cells (leukocytes) in whole blood, this may indicate that larger leukocytes in triploids contribute more to leukocrit values than the higher leukocyte numbers observed in diploids. This distinction should be considered when interpreting leukocrit as an indicator or immune status, particularly in response to pathogen infection.

In addition to the leukocyte count and leukocrit, we analyzed parameters of innate immunity, including respiratory burst, complement activity, and lysozyme concentration. As reported by Tolarová et al. (16), only respiratory burst, a rapid increase in oxygen consumption by immune cells such as neutrophils and macrophages important for combating pathogens, varied with ploidy status in tench. A study of turbot (*Psetta maxima* L., 1758) by Budiño et al. (72) found higher respiratory burst and phagocytic activity in triploid fish, although the number of neutrophils was higher in diploids, resulting in comparable respiratory burst activity and the phagocytosis per microliter of blood. Similarly, Chalmers et al. (73) showed that triploid Atlantic salmon exhibited higher respiratory burst activity but lower blood cell counts following furunculosis vaccination, suggesting that reduced cell numbers may be compensated by increased cellular activity. In contrast, a study of *Astyanax altiparanae* Garutti and Britski, 2000 showed that triploid induction led to reduced phagocytic capacity (67).

Our findings align with those of Budiño et al. (72) and Chalmers et al. (73), indicating that many components of the innate immune system function similarly in diploid and triploid tench, and that lower leukocyte counts in triploids may be offset by higher cellular activity. Differences in physiology, haematology, and immunity between triploid and diploid fish, including those reported in our study, may thus be explained by (1) increased heterozygosity of triploids, (2) a reduced number of larger cells in key organs (brain, retina, kidney, liver, testes, and ovaries) and tissues (blood, cartilage, muscle, epithelium), and (3) impaired gametogenesis as suggested by Benfey (2) and Maxime (74).

Although we anticipated associations between parasite infection and fish condition, haematological profile, physiology, and immunity in diploid and triploid tench, the observed relationships were generally weak. This may be due to overall low parasite abundance and species richness in tench. Moreover, significant seasonal and ploidy effects on parasite infection, along with variable responses among the four most common parasite species, may obscure correlations between overall parasite load and parameters linked to condition, physiology or immunity. Nonetheless, trends in associations between parasite infection and health-related traits were more pronounced in diploid tench than in triploids. This may be attributable to presumed higher heterozygosity of triploids, physiological differences related to cell size, and host–parasite coevolutionary interactions between diploid fish and their associated parasites. Reproductive investment, expressed by GSI, in both diploid and triploid females and males significantly correlated with total parasite abundance and with abundance of *A. tincae*. These correlations were stronger for diploids, suggesting a greater impact of this trematode parasite (likely weakened immunity) when increasing reproductive investment. This finding is consistent with previous studies on gonad development, which reported delayed or reduced gonadal growth in triploid tench (13, 30, 70).

Correlations between haematological and immune parameters and total parasite load or abundance of the four most frequent parasite species in tench were generally weak and often disappeared after correction for multiple testing. Therefore, these correlations should be interpreted with caution. Published studies on various fish species under different experimental conditions have yielded mixed results regarding immune function in diploid versus triploid fish. In some cases, triploid induction has been associated with immune impairments and reduced disease resistance as indicated by lower expression of immunity-related genes (IL-1 and TGF-β) in induced triploid *A. altiparanae* compared to diploids (67). A recent study by Jablonska et al. (75) found differences in the TLR gene expression between diploid and triploid sterlet (*Acipenser ruthenus* L., 1758), with TLRs being key components of the innate immune system responsible for recognizing pathogen-associated molecular patterns.

In contrast, other studies suggest that fish ploidy does not affect the pathogen susceptibility or innate serum immune responses. For example, no differences were found in the manifestation or severity of *Neoparamoeba perurans* Young, Crosbie, Adams, Nowak and Morrison, 2007 infection (causative agent of amoebic gill disease) in diploid and triploid Atlantic salmon (76). Similarly, a study on natural triploid (gynogenetic females) and diploid specimens of gibel carp (*Carassius gibelio*) showed that triploids were unaffected by parasite assemblage composition in terms of innate immunity and physiology (77). However, another study using *C. gibelio* under experimental infection with *Diplostomum pseudospathaceum* Niewiadomska, 1984 revealed a limited ability of triploid gynogenetic fish to cope with higher parasite infection, likely due to impaired immune gene activation (28). We propose that further studies on tench immunity should focus on differential gene expression and quantification of key immune genes, ideally under controlled experimental infections with a single parasite species, to clarify the role of ploidy in parasite susceptibility and resistance.

## 5 Conclusion

In conclusion, our study demonstrated that the ploidy status in tench affected metazoan parasite infection, reproductive investment (measured by GSI), haematological parameters, and immune function (specifically, leukocyte count and phagocyte activity expressed by respiratory burst). We revealed that triploids tended to have lower parasite load than diploids throughout the year. Nevertheless, our findings indicate that polyploidy exerts non-uniform effects on infection by different parasite species. Triploids exhibited lower abundance of the host-specific adult trematode *A. tincae*, yet significantly higher abundance of the larval cestode *V. campylancristrota*. Highly prevalent and moderately abundant monogenean specialist *G. tincae* was found in triploids and diploids at the similar intensity of infection. The other parasite species exhibited generally low infection parameters. We also identified an association between parasite infection and gonadosomatic condition, indicating that increased reproductive investment may elevate infection risk, particularly in diploid tench. Our results suggest potential effects of the three most abundant parasite taxa (*Gyrodactylus tincae*, *Asymphylodora tincae* and Myxosporea spp.) on tench immunity. The observed differences in parasite infection between triploid and diploid tench and the associations between parasite load and condition- and health-related traits may be explained by several factors: (1) presumed higher heterozygosity in triploids, (2) physiological differences related to cell size and number of cells in key organs and tissues, (3) variation in feeding performance, and (4) host–parasite coevolutionary interactions between diploid tench and their associated parasites.

## Data Availability

The raw data supporting the conclusions of this article will be made available by the authors, without undue reservation.
