# Supplementary material for: Susceptibility to metazoan parasite infection in amphimictic diploid and induced triploid tench (Tinca tinca L., 1758): the role of parasites in fish aquaculture
Source: Front Vet Sci. 2025 Nov 13;12:1686708. doi: 10.3389/fvets.2025.1686708 (PMC12661311; doi:10.3389/fvets.2025.1686708)
Supplement: Supplementary file 1 [file Table_1.docx]

Supplementary Table S1. Spearman correlation coefficients (r) and their respective *p*-values for correlation between abundance of the most frequent parasite species (*Asymphylodora tincae*, *Gyrodactylus tincae*, *Valipora campylancristrota*, Myxosporea spp.) and fish condition, physiological and immunological parameters, calculated separately for diploids (2n) and triploids (3n). Significant correlations (*p* < 0.05) are in bold, correlations that became non-significant after Boferroni correction (0.0033 > *p* < 0.050) are in bold italics.

*Asymphylodora tincae*

|  | r | | | *p*-value | |
| --- | --- | --- | --- | --- | --- |
|  | 2n | | 3n | 2n | 3n |
| Fulton´s condition factor | -0.054 | | 0.042 | 0.635 | 0.716 |
| HSI | **-0.424** | | ***-0.281*** | **0.000** | ***0.013*** |
| SSI | -0.016 | | ***0.223*** | 0.886 | ***0.048*** |
| GSI males | **0.493** | | ***0.405*** | **0.001** | ***0.009*** |
| GSI females | | **0.567** | **0.475** | **0.000** | **0.003** |
| Erythrocyte count | -0.033 | | ***0.248*** | 0.773 | ***0.031*** |
| Haematocrit | -0.054 | | ***0.230*** | 0.635 | ***0.044*** |
| Haemoglobin | ***-0.263*** | | 0.156 | ***0.018*** | 0.170 |
| Glucose | **-0.516** | | -0.187 | **0.000** | 0.098 |
| 11-ketotestosterone | 0.306 | | 0.142 | 0.055 | 0.376 |
| Leukocyte count | 0.207 | | 0.210 | 0.067 | 0.067 |
| Leukocrit | ***0.261*** | | ***0.254*** | ***0.029*** | ***0.037*** |
| Respiratory burst | 0.209 | | 0.053 | 0.084 | 0.644 |
| Complement activity | ***-0.249*** | | 0.109 | ***0.033*** | 0.375 |
| Lysozyme | ***-0.271*** | | -0.165 | ***0.015*** | 0.147 |

*Gyrodactylus tincae*

|  | r | | *p*-value | |
| --- | --- | --- | --- | --- |
|  | 2n | 3n | 2n | 3n |
| Fulton´s condition factor | 0.114 | 0.019 | 0.312 | 0.869 |
| HSI | **0.423** | ***0.269*** | **0.000** | ***0.017*** |
| SSI | 0.058 | 0.044 | 0.609 | 0.700 |
| GSI males | 0.294 | 0.304 | 0.062 | 0.054 |
| GSI females | -0.044 | -0.012 | 0.785 | 0.945 |
| Erythrocyte count | 0.024 | ***-0.265*** | 0.834 | ***0.021*** |
| Haematocrit | ***-0.222*** | **-0.470** | ***0.048*** | **0.000** |
| Haemoglobin | -0.102 | **-0.387** | 0.367 | **0.000** |
| Glucose | ***0.228*** | 0.097 | ***0.040*** | 0.395 |
| 11-ketotestosterone | -0.213 | -0.006 | 0.187 | 0.969 |
| Leukocyte count | ***-0.315*** | **-0.365** | ***0.005*** | **0.001** |
| Leukocrit | **-0.414** | -0.143 | **0.000** | 0.244 |
| Respiratory burst | **-0.365** | ***-0.239*** | **0.002** | ***0.036*** |
| Complement activity | -0.061 | 0.152 | 0.608 | 0.211 |
| Lysozyme | ***-0.259*** | -0.174 | ***0.021*** | 0.124 |

*Valipora campylancristrota*

|  | r | | *p*-value | |
| --- | --- | --- | --- | --- |
|  | 2n | 3n | 2n | 3n |
| Fulton´s condition factor | -0.034 | -0.099 | 0.761 | 0.385 |
| HSI | -0.184 | -0.050 | 0.101 | 0.665 |
| SSI | 0.012 | -0.041 | 0.915 | 0.719 |
| GSI males | 0.135 | ***0.345*** | 0.401 | ***0.027*** |
| GSI females | 0.278 | 0.223 | 0.082 | 0.179 |
| Erythrocyte count | 0.106 | 0.056 | 0.352 | 0.631 |
| Haematocrit | ***0.229*** | 0.172 | ***0.041*** | 0.135 |
| Haemoglobin | 0.106 | ***0.221*** | 0.345 | ***0.050*** |
| Glucose | -0.176 | 0.021 | 0.117 | 0.852 |
| 11-ketotestosterone | ***0.366*** | 0.120 | ***0.020*** | 0.455 |
| Leukocyte count | 0.111 | 0.056 | 0.331 | 0.631 |
| Leukocrit | 0.130 | 0.064 | 0.283 | 0.606 |
| Respiratory burst | -0.195 | 0.102 | 0.109 | 0.376 |
| Complement activity | -0.059 | 0.013 | 0.616 | 0.914 |
| Lysozyme | 0.102 | 0.013 | 0.369 | 0.908 |

Myxosporea spp.

|  | r | | *p*-value | |
| --- | --- | --- | --- | --- |
|  | 2n | 3n | 2n | 3n |
| Fulton´s condition factor | 0.160 | ***0.267*** | 0.153 | ***0.017*** |
| HSI | ***0.318*** | ***0.248*** | ***0.004*** | ***0.028*** |
| SSI | -0.007 | -0.074 | 0.952 | 0.516 |
| GSI males | 0.089 | 0.187 | 0.578 | 0.242 |
| GSI females | 0.016 | -0.095 | 0.923 | 0.571 |
| Erythrocyte count | -0.148 | 0.032 | 0.194 | 0.784 |
| Haematocrit | -0.219 | -0.100 | 0.051 | 0.386 |
| Haemoglobin | 0.022 | 0.175 | 0.845 | 0.124 |
| Glucose | ***0.277*** | **0.325** | ***0.012*** | **0.003** |
| 11-ketotestosterone | -0.158 | -0.302 | 0.330 | 0.055 |
| Leukocyte count | **-0.337** | **-0.429** | **0.002** | **0.000** |
| Leukocrit | -0.198 | ***-0.333*** | 0.100 | ***0.006*** |
| Respiratory burst | ***-0.323*** | -0.202 | ***0.007*** | 0.078 |
| Complement activity | -0.001 | -0.092 | 0.997 | 0.452 |
| Lysozyme | -0.170 | -0.198 | 0.132 | 0.081 |
